# Supplementary material for: The Hidden Story of Heterogeneous B-raf V600E Mutation Quantitative Protein Expression in Metastatic Melanoma—Association with Clinical Outcome and Tumor Phenotypes
Source: Cancers (Basel). 2019 Dec 9;11(12):1981. doi: 10.3390/cancers11121981 (PMC6966659; doi:10.3390/cancers11121981)
Supplement: Supplementary file 1 [file cancers-11-01981-s001.pdf]

cancers

MDPI

Supplementary information

Materials and Methods

Protein Extraction, Digestion and Automated C18 Desalting Workflow

Protein extraction was performed on sectioned, fresh-frozen human MM lymph node metastasis tissues (10 µm) using the Bioruptor plus, model UCD-300 (Dieagenode). A total of 56 MM tissue samples were lysed in 100 µL lysis buffer containing 4 M urea and 100 mM ammonium bicarbonate. After briefly vortexing, samples were sonicated in the Bioruptor for 40 cycles at 4 °C. Each cycle consisted of 15 s at high power and 15 s without sonication. The samples were then centrifuged at 10,000 × g for 10 min at 4 °C. A pool of MM lysates was also prepared as the reference sample. The protein content in the supernatant was determined using the colorimetric micro BCA Protein Assay kit (Thermo Fisher Scientific, Rockford, IL, USA).

Protein digestion was performed on the AssayMAP Bravo (Agilent Technologies, Lexington, MA, USA) platform using the digestion v2.0 protocol. Proteins were reduced with 10 mM DTT for 1 h at room temperature (RT) and sequentially alkylated with 20 mM iodoacetamide for 30 min in the dark at RT. To decrease the urea concentration, the samples were then diluted approximately seven times with 100 mM ammonium bicarbonate. Digestion was performed in two steps. Proteins were first incubated with Lys-C at a 1:50 (w/w) ratio (enzyme:protein) for 5 h, and then trypsin was then added at a 1:50 (w/w) ratio and the mixture incubated overnight at RT. The reaction was quenched by adding 20% TFA to a final concentration of ~1%. Peptides were desalted on the AssayMAP Bravo platform using the peptide cleanup v2.0 protocol. C18 cartridges (Agilent, 5 µL bed volume) were primed with 100 µL 90% acetonitrile (ACN) and equilibrated with 70 µL 0.1% TFA at a flow rate of 10 µL/min. The samples were loaded at 5 µL/min, followed by an internal cartridge wash with 0.1% TFA at a flow rate of 10 µL/min. Peptides were eluted with 30 µL 80% ACN, 0.1% TFA and dried in speed vac prior to TMT labeling.

TMT 11-Plex Labeling

The peptide content in each sample was determined using the quantitative colorimetric peptide assay kit (Thermo Fisher Scientific) to ensure equal amounts of material in each TMT channel. TMT labeling was performed according to manufacturer's instructions. Samples were resuspended in 100 µL of 200 mM TEAB and individual TMT 11-plex reagents were dissolved in 41 µL of dried ACN. Peptides were labeled by mixing the peptide solution with TMT 11-plex reagents for 1 h at room temperature. Reaction was quenched by adding 1 µL of 5% hydroxylamine and incubation at room temperature for 15 min. Then, the labeled peptides were mixed in a single tube, the volume was reduced in a speed vac, and the peptides were cleaned up using a C-18 Sep-Pak cartridge (Waters, Chromatography Europe, Etten-Leur, The Netherlands). The eluted peptides were dried in a speed vac and finally resuspended in 20 mM ammonium formiate prior to high pH fractionation.

Off Line High pH Fractionation

Each batch of TMT-11 labelled peptide was fractionated using a Phenomenex Aeris Widepore XB-C8 (3.6 µm, 2.1 × 100 mm) column on an 1100 Series HPLC (Agilent) operating at 80 µL/min. The mobile phases were solvent A: 20 mM ammonium formate and solvent B: 80% ACN-20% water containing 20 mM ammonium formate. Both solvents were adjusted to pH 10 with ammonium hydroxide. Separation was performed using the following gradient: 0 min 5% B; 1 min 20% B; 60 min 40% B; 90 min 90% B; 120 min 90% B. The column was operated at RT and the detection wavelength was 214 nm. Ninety-eight fractions were collected at 1 min intervals and further concatenated to 24 or 25 fractions, which were dried in a SpeedVac (Eppendorf).

nLC-MS/MS Analysis

nLC-MS/MS analysis was performed on an Ultimate 3000 HPLC coupled to a Q Exactive HF-X mass spectrometer (Thermo Scientific, San Jose, CA, USA). Peptides from each fraction (1 µg) were loaded onto a trap column (Acclaim1 PepMap 100 pre-column, 75 µm, 2 cm, C18, 3 mm, 100 Å, Thermo Scientific) and then separated on an analytical column (EASY-Spray column, 25 cm, 75 µm i.d., PepMap RSLC C18, 2 mm, 100Å, Thermo Scientific) using a 120 min ACN gradient with 0.1% formic acid at a flow rate of 300 nL/min and a column temperature of 45 °C. Q Exactive HF-X mass spectrometer was set using the TMT node, as follows: full MS scans at *m/z* 350–1400 with a resolution of 120,000 at *m/z* 200, a target AGC value of 3×10^6 and IT of 50 ms, DDA selection of the 20 most intense ions for fragmentation in HCD collision cell with an NCE of 34 and MS/MS spectra acquisition in the Orbitrap analyzer at a resolution of 45,000 (at *m/z* 200) with a maximum IT of 86 ms, fixed first mass of 110 *m/z*, isolation window of 0.7 Da and dynamic exclusion of 30 s.

Quality controls were introduced to evaluate and maintain the performance of the nLC-MS/MS systems. A protein digest from Hela cells (Pierce HeLa Protein Digest Standard, Thermo Fisher Scientific) mixed with a standard peptide mixture (Pierce Peptide Retention Time Calibration Mixture) was measured every 10 LC-MS/MS analysis. This allowed the monitoring of peak width, retention time, base peak intensity, number of MS/MS, PSMs, peptides and proteins identified, and MS/MS success rate, among other parameters.

Data Analysis

Data were processed with Proteome Discoverer 2.3 (Thermo Fisher Scientific) using the Sequest HT search engine. The search was performed against the Homo sapiens UniProt revised database (2018-10-01) and the B-raf V600E mutant protein sequence. Cysteine carbamidomethylation was set as a fixed modification, while methionine oxidation and TMT 6plex at peptide N-terminus and lysine were set as variable modifications: peptide mass tolerance for the precursor ions and MS/MS spectra were 10 ppm and 0.02 Da, respectively. A maximum of two missed cleavage sites was accepted and FDR were set at 0.01 for identification at peptide level. The PD software allowed the introduction of reporter ion interferences for each batch of TMT 11-plex reagents as isotope correction factors in the quantification method.

The search results were directly processed in Perseus software [1]. A filtering criterion was set to keep the identified proteins with the quantified values of all reporter ions (no missing value) in the final identification list. The protein intensities were log2–transformed and normalized by subtracting the median intensity in each sample. The relative abundance values were obtained by subtracting the intensity of the protein in the reference sample.

For statistical analysis, samples were separated in two groups according to the levels of mutated B-raf: Group 1 (V600E_H), with B-raf > 1.65, and Group 2 (V600E_H), B-raf ≤ 1.65. The cut-off value (1.65) was selected by an ROC curve, where the ability of mutated B-raf to discriminate between long and short survivals (considering a three years' survival) was analyzed (Figure S1). To make the ROC curve, patients of less than 40 years at the age of diagnosis were excluded. Kaplan–Meier survival analysis with log-rank, Breslow and Tarone Ware testing was used for univariate analysis between Groups 1 and 2. *P*-value < 0.05 was considered statistically significant. These analyses were performed using SPSS 25 (SPSS Inc, Chicago, IL, USA) software.

Differentially expressed proteins between V600E_H and 2 V600E_L were determined by Student t-test (two-tails). In this case, proteins quantified in at least three samples in each group were considered for the analysis and p-values < 0.01 dictated significant protein changes. Principal component analysis (PCA) was performed in R [2,3] ('FactoMineR' package) to visualize the behavior of DEPs. These proteins were included in the bioinformatics analysis, including hierarchical clustering ('pheatmap' R package; distance: Euclidean; linkage: average) and heat map generation. For functional analysis of the DEPs, the Ingenuity IPA Core Analysis was performed (Ingenuity, Qiagen).

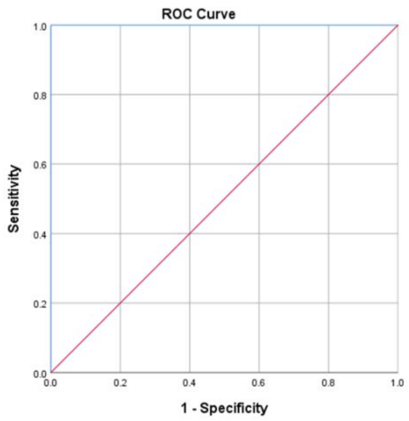

Figure S1. ROC curve analysis for the discrimination analysis between patients with long (more than three years) or short (less than three years) survival, according to B-raf V600E relative abundances measured by mass spectrometry on melanoma tumors. The cut-off (relative abundance of B-raf V600E higher than 1.65) was determined considering 100% of sensitivity and specificity for long survival patients.

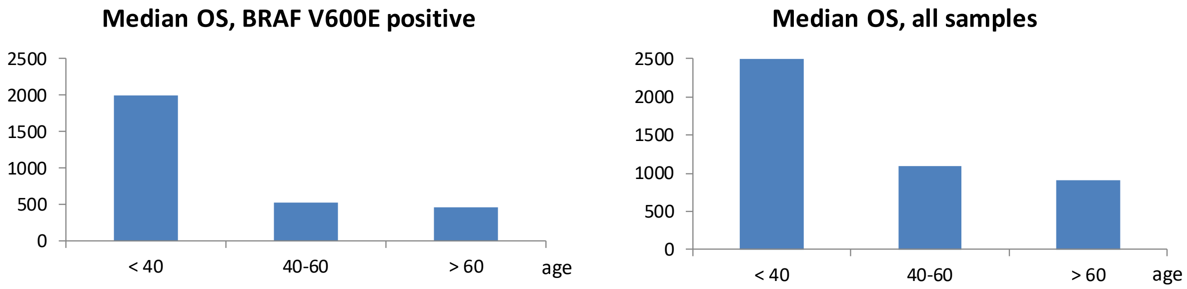

Figure S2. Bar graph of median overall survival for different age group of patients with B-raf V600E positive melanomas and for the whole sample set.

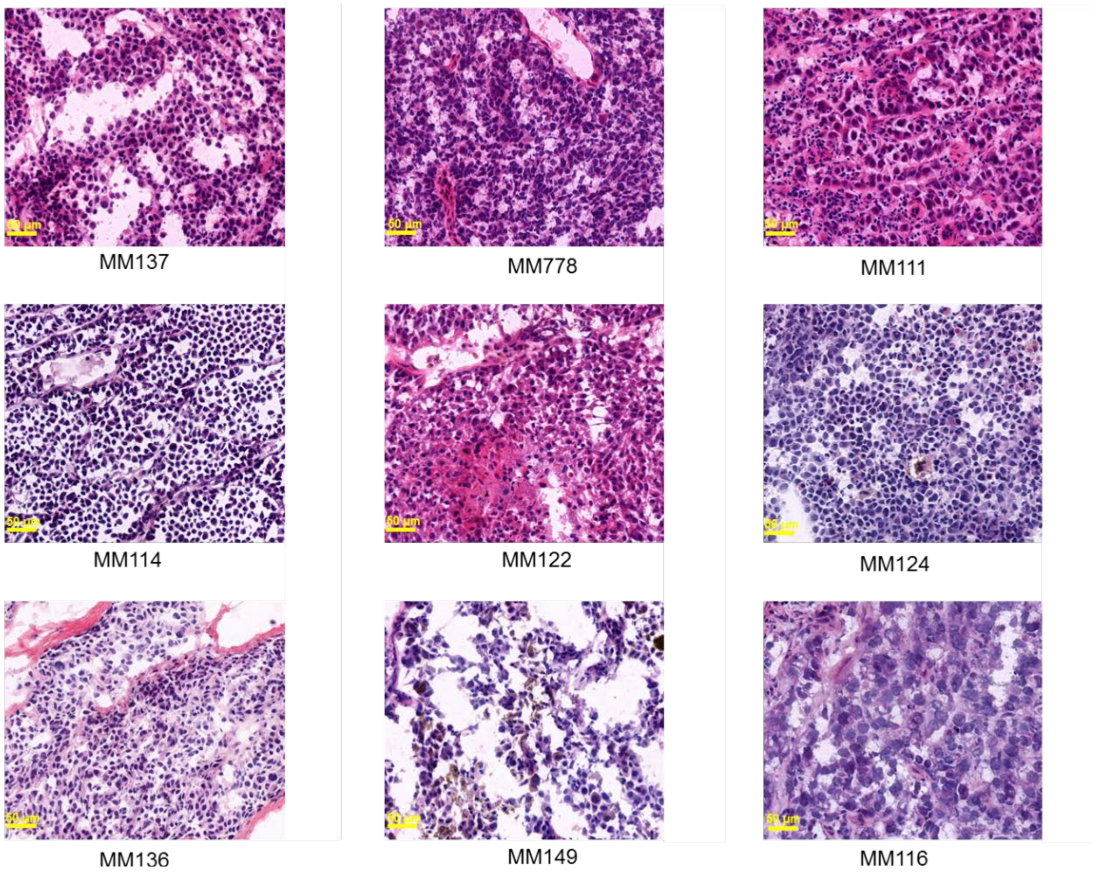

High-expressing B-raf V600E tumors.

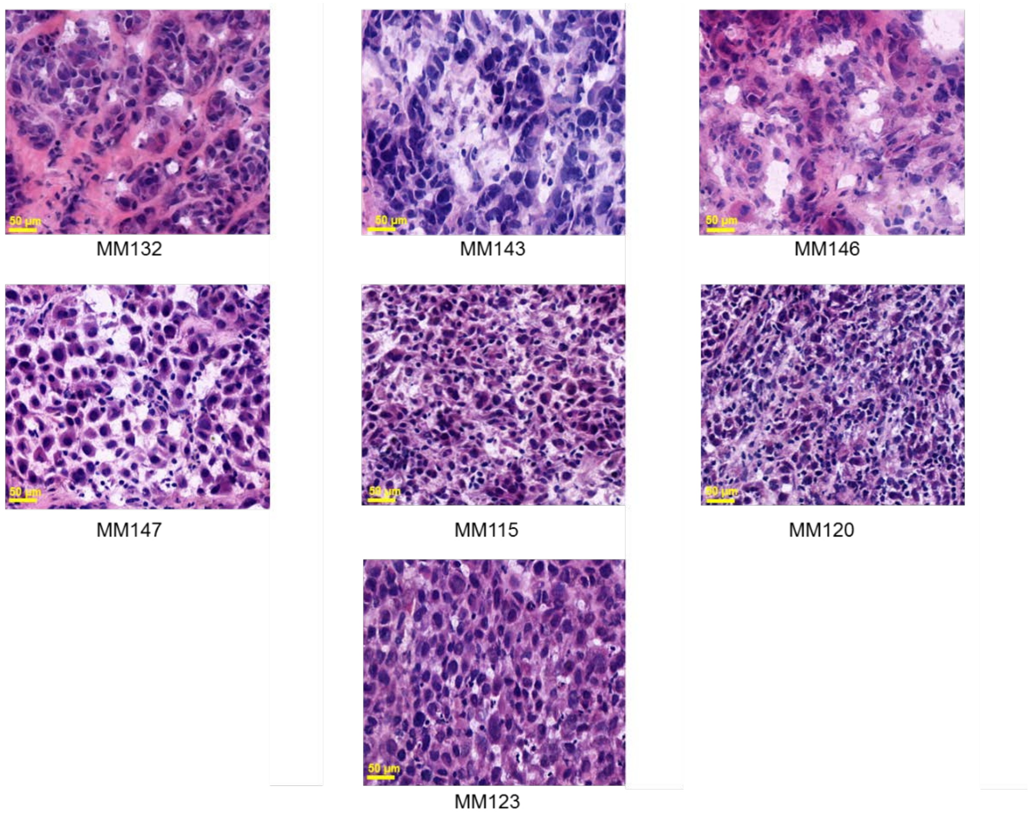

Low-expressing B-raf V600E tumors.

Figure 3. Histological images of mutation-positive metastatic melanoma samples. For all the images the magnification and scale were 10× and 50 µm, respectively.

Table S1. Patient clinical data, tumor content and BRAF status results for mutation-positive melanoma metastases.

#	Sample	% Tumor	Gender	Stage	Metastasis	Age.Diag	Os.Days	Dead/Alive	BRAF Status (DNA)	BRAF Status (RNA)	BRAF Status Status (MS)	BRAF V600E (MS Guatitation)	
1	MM149	91.6	Male	3	Subcutaneous	86	89	dead	V600E	V600E	V600E	6.165	
2	MM111	99.0	Female	4	Subcutaneous	50	368	dead	N/A	V600E	V600E	4.744	
3	MM136	85.0	Male	3	Lymph node	65	453	dead	V600E	V600E	V600E	3.857	
4	MM114	80.5	Female	4	Lymph node	52	126	dead	V600E	V600E	V600E	3.396	High
5	MM137	82.6	Male	3	NA	59	476	dead	V600E	V600E	V600E	3.338	expression
6	MM109	97.0	Female	3	Lymph node	29	1479	alive	WT	V600E	V600E	2.829	group
7	MM130	95.5	Male	3	Lymph node	39	2530	dead	V600E	V600E	V600E	2.076	
8	MM122	91.3	Male	4	Visceral	46	66	dead	V600E	V600E	V600E	1.881	
9	MM124	97.5	Male	3	Lymph node	60	523	alive	V600E	V600E	V600E	1.844	
10	MM133	95.0	Female	3	Lymph node	24	5005	alive	V600E	V600E	V600E	1.772	
11	MM138	96.0	Male	4	Lymph node	72	42	dead	V600E	V600E	V600E	1.772	
12	MM116	15.0	Male	4	Lymph node	69	93	dead	V600E	V600E	V600E	1.701	
13	MM146	88.4	Male	4	Lymph node	65	1455	dead	V600E	V600E	V600E	1.613	
14	MM143	95.4	Male	3	Lymph node	55	2800	alive	V600E	V600E	V600E	1.607	
15	MM147	85.9	Male	3	Subcutaneous	65	1222	dead	V600E	V600E	V600E	1.593	Low
16	MM120	98.0	Female	3	Lymph node	46	652	alive	WT	V600E	V600E	1.491	expression
17	MM105	71.3	Male	3	Lymph node	77	1097	dead	WT	WT	V600E	1.392	group
18	MM115	94.8	Female	3	Lymph node	73	1035	alive	V600E	V600E	V600E	1.337	
19	MM154	83.0	Male	3	Lymph node	39	1329	dead	V600E	V600E	V600E	1.31	
20	MM123	76.5	Male	3	Lymph node	59	307	alive	V600E	V600E	V600E	1.278	
21	MM118	82.0	Male	4	Lymph node	72	789	alive	WT	WT	V600E	1.023	
22	MM132	79.3	Male	3	Lymph node	69	3582	dead	V600E	V600E	V600E	1.009	

age. diag: age at diagnosis; os.days: overall survival; NA: Not analyzed; ND: No data available.

Table S2. Patient clinical data and WT BRAF detection by different techniques.

#	Sample	% Tumor	Gender	Stage	Metastasis	Age.Diag	Os.Days	Dead/Alive	BRAF Status	BRAF Status	BRAF Status
									(DNA)	(RNA)	Status (MS)
23	MM101	0.0	Male	4	Lymph node	89	638	dead	WT	WT	WT
24	MM102	76.3	Male	3	Lymph node	72	849	dead	WT	WT	WT
25	MM103	96.6	Male	4	Subcutaneous	68	392	dead	WT	WT	WT
26	MM104	79.5	Female	3	Lymph node	62	1779	alive	WT	WT	WT
27	MM106	92.8	Male	4	Lymph node	57	542	dead	WT	WT	WT
28	MM107	82.8	Male	3	Lymph node	82	574	dead	WT	WT	WT
29	MM108	96.0	Female	3	Lymph node	70	1406	alive	WT	WT	WT
30	MM110	1.0	Male	3	Lymph node	60	1260	alive	WT	WT	WT
31	MM112	99.0	Female	3	Lymph node	73	1379	alive	WT	N/A	WT
32	MM113	90.3	Male	3	Lymph node	70	399	dead	WT	WT	WT
33	MM117	99.0	Female	3	Lymph node	62	303	dead	WT	WT	WT
34	MM119	85.0	Male	3	Lymph node	75	970	dead	WT	N/A	WT
35	MM121	0.0	Male	1	Cutaneous	77	170	dead	WT	WT	WT
36	MM125	98.0	Female	3	Lymph node	70	424	alive	WT	WT	WT
37	MM126	0.0	Male	4	Lymph node	62	318	alive	WT	WT	WT
38	MM127	0.0	Male	4	Lymph node	65	165	dead	V600E	WT	WT
39	MM128	58.0	Female	3	Lymph node	54	174	alive	WT	NA	WT
40	MM129	90.0	Male	3	Lymph node	53	642	dead	WT	WT	WT
41	MM131	83.3	Male	3	Lymph node	68	6343	alive	WT	WT	WT
42	MM134	86.3	Female	3	Lymph node	74	461	dead	WT	WT	WT
43	MM135	73.0	Male	3	Lymph node	71	336	dead	WT	WT	WT
44	MM139	76.1	Male	4	Lymph node	61	275	dead	WT	WT	WT
45	MM140	61.3	Female	3	Lymph node	57	3363	alive	WT	WT	WT
46	MM141	48.7	Male	3	Lymph node	50	3206	alive	WT	WT	WT
47	MM142	82.1	Male	4	Lymph node	70	50	dead	WT	WT	WT
48	MM144	65.1	ND	ND	ND	ND	ND	ND	N/A	WT	WT

#	Sample	% Tumor	Gender	Stage	Metastasis	Age.Diag	Os.Days	Dead/Alive	BRAF Status (DNA)	BRAF Status (RNA)	BRAF Status Status (MS)
49	MM145	93.7	Male	1	Subcutaneous	53	511	dead	WT	WT	WT
50	MM148	98.0	Male	4	Visceral	63	2619	alive	WT	WT	WT
51	MM150	74.7	Male	3	Lymph node	55	2574	alive	WT	WT	WT
52	MM151	62.3	Female	4	Lymph node	80	386	dead	WT	WT	WT
53	MM152	97.0	Male	3	Lymph node	70	56	dead	WT	WT	WT
54	MM153	95.3	Male	4	ND	65	279	dead	WT	WT	WT
55	MM155	0.0	Male	3	Lymph node	75	519	dead	N/A	N/A	WT
56	MM156	98.0	Female	3	Lymph node	76	1401	dead	WT	WT	WT

age. diag: age at diagnosis; os.days: overall survival; NA: Not analyzed; ND: No data available.

Table S3. Co-isolation interference in every TMT 11-plex batch for the mutated peptide of the BRAF V600E protein.

TMT Batch #	1	2	3	4	5	6
**Isolation Interference (%)**	27	41	0	20	0	0
